# School Counselors’ Perspectives of a Web-Based Stepped Care Mental Health Service for Schools: Cross-Sectional Online Survey

**DOI:** 10.2196/mental.8369

**Published:** 2017-11-20

**Authors:** Bridianne O'Dea, Catherine King, Mirjana Subotic-Kerry, Kathleen O'Moore, Helen Christensen

**Affiliations:** ^1^ Black Dog Institute Sydney Australia; ^2^ Faculty of Medicine University of New South Wales Sydney Australia

**Keywords:** secondary schools, adolescent, counseling, internet, depression

## Abstract

**Background:**

Mental health problems are common among youth in high school, and school counselors play a key role in the provision of school-based mental health care. However, school counselors occupy a multispecialist position that makes it difficult for them to provide care to all of those who are in need in a timely manner. A Web-based mental health service that offers screening, psychological therapy, and monitoring may help counselors manage time and provide additional oversight to students. However, for such a model to be implemented successfully, school counselors’ attitudes toward Web-based resources and services need to be measured.

**Objective:**

This study aimed to examine the acceptability of a proposed Web-based mental health service, the feasibility of providing this type of service in the school context, and the barriers and facilitators to implementation as perceived by school counselors in New South Wales (NSW), Australia.

**Methods:**

This study utilized an online cross-sectional survey to measure school counselors’ perspectives.

**Results:**

A total of 145 school counselors completed the survey. Overall, 82.1% (119/145) thought that the proposed service would be helpful to students. One-third reported that they would recommend the proposed model, with the remaining reporting potential concerns. Years of experience was the only background factor associated with a higher level of comfort with the proposed service (*P*=.048). Personal beliefs, knowledge and awareness, Internet accessibility, privacy, and confidentiality were found to influence, both positively and negatively, the likelihood of school counselors implementing a Web-based school mental health service.

**Conclusions:**

The findings of this study confirmed that greater support and resources are needed to facilitate what is already a challenging and emotionally demanding role for school counselors. Although the school counselors in this study were open to the proposed service model, successful implementation will require that the issues outlined are carefully addressed.

## Introduction

### Background

Worldwide, mental illness in adolescence is a major public health burden. Up to 50% of all mental disorders emerge before the age of 18 [1] and are associated with poor academic performance, early termination of schooling, elevated suicide risk, and comorbidities such as substance misuse and self-harm [2-4]. Despite the substantial impact, professional help-seeking is low [5-8]. When help is sought, adolescents prefer informal sources who are familiar and trusted [9]. Barriers to formal help-seeking include poor mental health literacy, perceived negative stigma [10,11], and service inaccessibility [12]. Current youth services are overburdened, fragmented, and unable to provide preventative care for the wide spectrum of youth presentations [13]. Given that up to 70% of those who experience mental illness in adolescence will relapse within 5 years, prevention and early intervention is key [14,15]. There is a demonstrated need to improve the ability to screen, initiate prevention, and respond to youth at all levels of severity of mental ill health [13].

Schools play a vital role in fostering the social and emotional well-being of youth [16-18]. Schools have a formalized duty of care, defined as the moral and legal obligation to ensure the well-being of their students. In addition to this, schools are an ideal setting to promote mental health as adolescents spend a significant portion of their time at school; schools are a natural setting for learning skills and strategies; mental health education can be integrated into the curriculum (reducing time, location, and cost barriers); access to parents and families is easily facilitated; and the social context allows for stigma reduction [19]. School staff are well positioned to identify changes in students’ behavior and to identify threats to well-being in the school environment [20]. As attendance at school is typically compulsory until the age of 16 years, schools give unparalleled access to youth [21]. Despite this, secondary schools remain underutilized for the prevention and treatment of mental health problems.

In Australia, approaches to mental health in secondary schools are varied and multifaceted. In the state of New South Wales (NSW), student mental health is mainly supported by a school counselor who is employed to offer counseling [17]. All NSW students can expect to have access to a school counselor, although recent figures have shown that the student-to-counselor ratio is double the recommendation of 500:1 [17,22]. This disparity is also common in the United States [23], which may reduce the school counselors’ capacity to proactively identify students in need [12]. Australian school counselors also occupy a multispecialist role as they conduct psychometric assessments, deliver training to staff, and liaise with parents, teachers, and external services [24]. These demands can lead to a sense of role ambiguity [24]. Despite the emotional impacts of the role, school counselors can feel isolated because of confidentiality issues [25,26] and a perceived lack of clinical supervision [26]. Studies in the United States have found school counselors experience high levels of stress and burnout due to role conflict, professional overload, and lack of autonomy [27,28]. It is likely that these factors also affect Australian school counselors and their capacity to provide care, although comprehensive studies among this population are lacking. Although school counselors are often supported by a broader range of school programs, there is substantial variability in the type, extent, quality, and evidence base of these [29-31]. Competing responsibilities, parent disengagement, lack of support from school staff [31], limited resources, and significant variations in staff training [32] have hindered implementation. As a result, many schools have a highly reactive, disjointed, and piecemeal approach to mental health [33] that lacks a universal and preventative focus [34].

Web-based mental health resources, also known as e-mental health, may overcome many of the challenges faced by school counselors [35]. Young people are very willing to seek help for mental health via the Internet [36,37], and Web resources provide convenient access to information and support. However, school counselors have been found to be reluctant to use some types of Web resources, specifically online counseling [36,38]. Lack of training, misbeliefs about quality and efficacy, confidentiality, and dishonesty concerns have been found to affect the use of online counseling in school settings [36,39]. It is likely that these barriers influence the uptake of other Web-based programs and resources, although there is little research examining school counselors’ perspectives toward more recent developments such as mobile technology, e-monitoring tools, automated feedback, stepped care, and interventions delivered to entire student populations at once. One such advance is *Smooth Sailing*—a Web-based mental health service developed by the Black Dog Institute, which is based on the principles of stepped care [35]. Delivered in the classroom using a mobile or desktop device, this service utilizes the Internet to triage students’ mental health and assist school counselors in identifying and attending to those in need. On the basis of their responses to clinically validated measures, students are allocated to one of five sequential treatment steps. The service offers low-intensity interventions (eg, Web-based psychoeducation and cognitive behavioral therapy) to youth with mild to moderate symptoms (ie, steps 0, 1, 2), whereas more costly, intensive face-to-face interventions (eg, school counseling) are reserved for those who are more severe (ie, steps 3, 4). Students who report suicidal ideation trigger an automatic email alert to the school counselor who responds within 48 hours, conducting the risk assessment and external referral. Although complex, this novel service may improve school counselors efficiency and capacity to prevent mental illness in students by detecting symptoms early [33]. Web-based programs are easily integrated into the service, as they are fully automated, acceptable to youth, preserve fidelity of care, and allow for ongoing monitoring and automated feedback [40,41]. This service is yet to be implemented in NSW schools, as the attitudes of school counselors toward this new type of care are not yet known.

### Objectives

Using an online survey, this study aimed to examine the acceptability of the proposed Web-based mental health service, the feasibility of providing this type of service in the school context, and the barriers and facilitators to implementation as perceived by school counselors in NSW, Australia. This study was guided by the Stage Based Implementation Framework [42] and the Medical Research Council’s Guide for Complex Interventions [43], which outline the need for stakeholder engagement in measuring the barriers and facilitators to implementation. Using these frameworks, this study aimed to bridge the research-to-practice gap that currently exists within school-based mental health care by consulting closely with school counselors as to their needs and capacity, context characteristics, and their willingness to integrate novel interventions into their practice. These findings will assist health service researchers who wish to leverage the Web to deliver efficient mental health services, at scale, and within the school setting.

## Methods

### Study Design

This study utilizes a cross-sectional online survey. Ethics approval was obtained from the University of New South Wales (approval: HC15456) and the NSW Department of Education (SERAP approval: 2015369).

### Sample Size

The NSW Department of Education estimates that there are 790 school counselors across NSW [17]. On the basis of this figure, and adopting a CI of 95% and margin of error of 7%, the sample size necessary for representativeness was calculated to be 157.

### Participants, Procedure, and Recruitment

Participants were aged over 18 years, fluent in English, and currently working as a school counselor in an NSW secondary school. Recruitment took place between July and October 2016 (108 days) via the Black Dog Institute website, social media channels, word of mouth, and an electronic direct mail-out to professional networks. The online survey link was included in all recruitment material such that participants were automatically directed to the study website. Online consent was provided before answering the survey questions, in which participants were required to confirm their status as a secondary school counselor in NSW. The survey was voluntary and could be completed on either a mobile or a computer device. Participants did not receive any financial benefit for their participation.

### Survey

The online survey is included as Multimedia Appendix 1. The questions were taken from a semistructured interview schedule and adapted for online delivery. The survey consisted of 91 questions, displayed over 17 pages, and took approximately 45 minutes to complete. Participants were not able to change their answers once saved and submitted. Participants’ Internet Protocol addresses were collected and checked for duplicates. The quantitative data consisted of all nominal and interval questions, whereas the qualitative data consisted of the free-text response options.

#### Demographics

A total of 12 questions assessed participant demographics and background factors, including age, gender, Aboriginal or Torres Strait Islander (ATSI) identification, place of birth, relationship status, highest level of education, registration status (registered or unregistered psychologist), years working as a school counselor, and number of schools currently working in.

#### Current Role

A total of six questions assessed the current school counselor role, including the type of school (funding: public or private, gender: single sex or coeducational), employment (part-time or full-time), school size (number of students enrolled), and school location (metropolitan or regional and rural). Using multiple-choice responses, participants were asked about the common referral pathways for students (self-referral, school counselors, teacher, year advisor, parent, and other), perceived barriers to care among students (stigma, low mental health literacy, personal embarrassment or shame, distrust in school, cultural perceptions, personal characteristics of counselor, confidentiality concerns, and other), and perceived prevalence of mental health issues. Workload questions related to average number of session per day, number of sessions related to mental health, mental health issues most prevalent, and a breakdown of daily tasks. Using a 5-point Likert scale ranging from not at all (1) to entirely (5), participants were asked how manageable their workload was and to what extent work stress impacted on their health and well-being. Participants were also asked whether they had ever experienced burnout (yes, no, unsure, rather not say). Frequency of providing personal contact details, frequency of providing care outside of school hours, frequency of deliberate self-care, and frequency of external service referral and parent engagement were also assessed (answered never, rarely, sometimes, often, always). Also using a 5-point Likert scale ranging from not at all (1) to entirely (5), participants were asked to rate how supported they felt by external services, parents, and other teachers. Participants were also asked how satisfied they were with the way their current school addressed the mental health of students, ranging from not at all (1) to entirely (5).

#### Openness to Practice

This was assessed using the Openness to Practice subscale from the Evidence-Based Practice Attitude Scale (EBPAS) [44,45]. This subscale is designed for mental health service providers and consists of four items in which respondents are asked to rate how likely they are to use or try different types of therapies and interventions. Answers are given using a 5-point Likert scale ranging from not at all (0) to a very great extent (4). Computing the overall mean creates the total score. Total scores range from 0 to 4, with higher scores indicating more favorable attitudes. In a comparative sample of mental health service providers working with children and adolescents, the mean openness score was found to be 2.49 (standard deviation [SD] 0.75). The internal consistency and reliability of this subscale was high for this sample (alpha=.78-.81).

#### Use of Web-Based Programs and Resources

Participants were asked to indicate how often they used, recommended, or referred students to (1) information websites, (2) online peer support, (3) mobile phone apps, (4) symptom-focused programs, (5) online counseling, and (6) telepsychiatry/ videoconferencing (answered never, sometimes, often, always). Responses were then dichotomized into “frequently recommended” (often and always) and “rarely recommended” (sometimes and never).

#### Attitudes Toward Smooth Sailing Service

The survey included short descriptions of the Smooth Sailing service to familiarize participants with the core components. As Smooth Sailing has not yet been publicly released, no school counselors were using this service at the time of the study. Participants were asked 13 questions about their level of comfort with certain features of the service (eg, referral pathways, provision of personal information, data access, computer-assisted decision making, risk management, feasibility of responding in 48 hours, short message service (SMS), and email monitoring). These were answered on a 5-point Likert scale ranging from not at all comfortable (1) to entirely comfortable (5). Short answer responses allowed participants to elaborate on reasons why or why not. Lastly, participants were asked whether they thought the service would be helpful to students (yes, no, unsure) and whether they would prefer that the service was offered outside of the school setting (yes, no, unsure).

### Data Analysis

Data were exported from Key Survey version 8.13, an online survey tool developed by WorldAPP, and analyzed using IBM SPSS Statistics version 22. Only those participants who completed the entire survey were included in the analyses. Basic descriptions were calculated and reported for demographics and role characteristics. To examine the factors associated with the level of comfort recommending the Smooth Sailing service, a series of chi-square tests and simple point-biserial correlations were computed. The aim of these analyses was to determine whether age, gender, years of experience, number of students within the school, registration as a psychologist, openness to practice, or manageability of workload were associated with acceptability. The free-text data were analyzed using Braun and Clarke’s [46] thematic analysis guidelines. This was an iterative process of reading and coding response to extract key themes and meanings. The answers were analyzed and classified into main themes using an initial coding framework established by one researcher (BOD). A second researcher (MSK) then used this framework to independently code the responses. Initial coder agreement was 88%, with final themes decided upon by consensus.

## Results

### Participants

A total of 145 (145/166, 87.3%) school counselors completed the survey in its entirety. Of these participants, the mean age was 43 years (SD 12.0, range: 24-71), 85.5% (124/145) were female, 1.4% (2/145) reported to be ATSI, and 84.1% (122/145) were born in Australia. The majority (77.2%, 112/145) were married or partnered. In terms of their education, 77.2% (112/145) had received postgraduate training and 61.4% (89/145) were registered as a psychologist with 15.9% (23/145) currently undertaking their registration process. A total of 75.9% (110/145) were working full-time. On average, participants reported that they had been a school counselor for 10 years (SD 13.0, range: 1-38 years) and in their career lifetime, had worked at 11 different schools (SD 13.0, range: 1-12). At the time of survey completion, 31.7% (46/145) were working at only one school, with the remainder based at two or more schools. The average school size was 790 students (SD 396.0, range: 21-2015), and 49.7% (72/145) of participants were working in a metropolitan area. A total of 56.6% (82/145) were working in a coeducational, publicly funded school.

### Current Role

On average, participants conducted 5 individual student sessions per day (SD 1.60, range: 2-18). When asked to select what proportion of their workload was dominated by various activities, individual therapy (45.1%), administration (19.3%), and psychometric assessments (13.9%) were the three highest responses. Crisis management (7.3%), risk assessments (6.9%), personal or group supervision (5.1%), and professional development (5.4%) occupied less of the participants’ workload. A total of 67.6% (98/145) reported that most of their sessions were related to mental health issues. The prevalent mental health issues were anxiety (92.4%, 134/145), depression (85.5%, 124/145), family (77.2%, 112/145), self-harm (55.1%, 80/145), and relationships (54.5%, 79/145).

The most common methods of gaining access to the school counselor were referrals from students themselves (36.6%, 53/145), year advisors (26.9%, 39/145), teachers (21.4%, 31/145), and other school staff (9.6%, 14/145). Direct initiation from the school counselor accounted for only 2.8% (4/145) of students’ access. When asked how comfortable they thought students were in approaching them about mental health, only 1 participant reported not at all, with 96.6% (140/145) feeling that students were somewhat to entirely comfortable seeking their help. “Perceived stigma from others” (64.8%, 94/145), “a lack of understanding or knowledge about school counseling” (62.8%, 91/145), “personal embarrassment or shame” (55.9%, 81/145), “wanting to remain anonymous” (38.6%, 56/145), “concerns about confidentiality” (37.2%, 54/145), “cultural perceptions about mental illness” (35.9%, 52/145), and “distrust in the school” (21.4%, 31/145) were identified as the main barriers inhibiting students from seeking school counselors’ help.

A total of 2.8% (4/145) reported that they had, at some point, provided their personal contact details to students, and one-third (32.4%, 47/145) had found themselves providing care to students outside of school hours. A total of 68.3% (99/145) often referred students to external services, with 30.3% (44/145) feeling entirely or moderately supported by these services. A total of 50.3% (73/145) often engaged with parents, with 37.2% (54/145) feeling entirely or moderately supported by them. Two-thirds of the sample (61.4%, 89/145) felt supported by other teachers. One-third (44/145) reported that their current workload was not at all (11.0%) or only slightly (19.3%) manageable. Two-thirds (66.9%, 97/145) reported that work stress impacted on their health and well-being, and 44.8% (65/145) reported that they had experienced burnout. Overall, less than half (47.6%, n=69/145) were moderately or entirely satisfied with the way their current school addressed mental health.

**Table 1:** Openness to practice scores.

Item	Mean (SD)	Those who answered great or very great extent (N=145) n (%)
I like to use new types of therapies/interventions to help my clients.	2.18 (0.70)	41 (28.3)
I am willing to try new types of therapies/interventions even if I have to follow a treatment manual.	2.42 (0.81)	64 (44.1)
I am willing to use new and different types of therapies/interventions developed by researchers.	2.70 (0.80)	88 (60.7)
I would try a new therapy/intervention even if it were different to what I am used to doing.	2.63 (0.85)	78 (53.8)

**Table 2:** Participants’ level of comfort with the features of the Web-based mental health service.

Feature	Mean (SD)
Students being sent emails and SMS to monitor their mental health	3.79 (1.04)
Students answering questions about their mental health over the Web	3.50 (0.99)
Students’ receiving additional psychological support from an external psychologist over the phone	3.44 (0.98)
Students receiving treatment for mental health via a Web-based service	3.39 (0.89)
Students providing personal details over the Web	3.06 (1.11)
Students’ mental health being assessed and classified by an automated Web-based service	3.04 (0.98)

### Openness to Practice

Overall, the sample yielded a mean openness score of 2.50 (SD 0.67), with 28% being open to trying new therapies or interventions to a great or very great extent. Table 1 outlines the mean scores for each item and the percentage who reported being open to a great or very great extent.

### Use of Web-Based Mental Health Resources

A total of 77.2% (112/145) of the sample used, recommended, or referred students to at least one type of Web-based resource or program, with these participants using an average of 2.24 different Web resources with students (SD 1.22). Among this group, psychoeducation websites (77.7%, 87/112) and mobile phone apps (75.0%, 84/112) were the most frequently used. The least frequently used were online counseling (31.3%, 35/112), automated symptom-reduction/ psychological therapy programs (21.4%, 24/112), peer support sites (16.1%, 18/112), and telepsychiatry/ videoconferencing (2.7%, 3/112).

### Level of Acceptability of the Proposed Service

Overall, one-third of the sample (n=44/145) reported that they were entirely comfortable recommending a student try a Web-based service for their mental health (mean 3.90, SD 0.95), with only 1 participant reporting not at all. Table 2 outlines participants’ mean levels of comfort with the different features of the proposed service.

Overall, a total of 82.1% (119/145) reported that the proposed service would be helpful to high school students, although 14.5% (21/145) preferred that this type of service was not offered in the school setting. A total of 26.9% (39/145) believed that responding to automated at-risk alerts (ie, initiating contact and conducting a face-to-face session for those at higher steps or with suicidality) within 48 hours would not currently be feasible. Only 7.6% (11/145) were entirely comfortable with teachers being involved in referring or recommending a Web-based mental health service to students.

### Factors Influencing Acceptability

Level of comfort with referring a student to a Web-based service was found to be significantly associated with years of experience (*r*=.17, *P*=.048). Participants’ age, school size, workload manageability, and openness to practice were not significantly associated with their level of comfort referring to a Web-based service (*r*=.09 to .17, *P*=.17-.88). Gender was also not significantly associated with level of comfort (*t*_1,143_=−1.50, *P*=.14), and nor was registration as a psychologist (*t*_1,143_=1.36, *P*=.18).

Level of comfort was found to be influenced by the following:

#### Knowledge, Familiarity and Confidence With Online Services

This theme encompassed school counselors’ knowledge and awareness of Web-based mental health services, their familiarity with service content, their overall confidence in the service, and their personal preferences for providing care. A total of 34% (n=50) reported that these aspects influenced their likelihood of referral or recommendation. One participant reported:

I need to have knowledge of and feel comfortable with a service prior to my recommending it.

Participant 31

Another participant stated:

...some hesitation in referring to new things due to lack of knowledge...I would really need to use it myself before recommending it.

Participant 90

One participant said:

I would only refer if I had a full understanding of it.

Participant 106

A portion of respondents (9%, n=13) also revealed that they simply had a personal preference for face-to-face care:

I don’t see it being engaging and worthwhile, the relationship is not the same.

Participant 103

It’s just not part of my practice.

Participant 109

#### Internet Accessibility

This theme related to school counselors’ perceptions that the Internet can generally lead to greater access to care, with 30% (n=44) reporting that accessibility issues influenced their likelihood of recommending or referring. Within this, 18% (n=26) felt the Internet improved access to care as it defied time and spatial barriers.

One participant stated:

Accessibility, cost effective, suits the student in regard to time and commitments, the right fit can provide a more timely response.

Participant 98

Another participant reported:

Rural and remote location—often there are no other options.

Participant 114

A subset (12%, n=18) said that they were unlikely to refer or recommend a Web-based service because some students might not have Internet access at home (n=15) or that the literacy levels of their students was not adequate (n=3). For example, one participant 14 stated:

I work in a low SES area and some of the families do not have Internet or can't afford to keep the Internet for long periods of time.

Participant 14

Another participant reported:

My most disadvantaged youth don't have sufficient credit on their phone or on home computer to access...they don't like to use these services in a public section of the school where they may be observed.

Participant 47

#### Beliefs About the Effectiveness and Accuracy of Web-Based Services

This theme referred to the beliefs, both positive and negative, that school counselors have about the effectiveness and clinical accuracy of Web-based mental health care. A total of 29% (n=43) reported that their beliefs about clinical effectiveness influenced the likelihood of referring or recommending a Web-based service. An example of one negative belief was “I believe face-to-face interventions are more effective,” whereas examples of positive beliefs were “it can monitor their health and provide support when I am not there” (n=20), “it complements the work I do with students” (n=17), and “it can help students feel that they are taking control” (n=6). Some respondents were concerned with the reliability and validity of the automated decision-making processes within the Web-based service, mainly concerned that young people will not be honest in their disclosure of symptoms. For example, one participant stated:

...a computer cannot decide when a young person is being truthful.

Participant 19

Participant 144 described the clinical classification system as “too binary.” A total of 38% (n=56) identified that personal beliefs about effectiveness and accuracy were likely to be a risk to successful service implementation.

#### Duty of Care

This theme related to school counselors’ concerns that a Web-based mental health service would complicate their duty of care, with 46% (n=66) feeling their capacity was diminished when students were engaged in a Web-based service. Some reported hesitation about referral. For example, one of the participants stated:

I feel that it is my job to see them.

Participant 44

Others were concerned with risk management:

There needs to be more than one person who knows the student is at-risk...the counselor might not get the message.

Participant 4

Another participant stated:

...the biggest risk is promising access to a school counselor within such a short time...my referral list is so long I just don't know how I could do it.

Participant 9

When asked what should happen when a student was not improving in a Web-based service, 56% (n=81) said the student should be referred to an external mental health professional, and 26% (n=37) felt that parents should be informed. When asked what should happen when a student is suicidal, 48% (n=70) said the student should be contacted by an external mental health professional, 33% (n=48) said the student’s parents should be contacted, and 26% (n=37) said emergency services should be contacted. Other suggestions included informing the school, offering e-crisis support services, and referring to the general practitioner (GP). However, participants expressed concerns with multiple caregivers:

...it needs to be very clear who is responsible for following up with each child.

Participant 10

...if collaboration between school and external provider does not work well, there are risks to client safety.

Participant 12

...things get more difficult with online treatment and monitoring when a student is found to be at risk as the process of contacting people that can keep them safe is more convoluted.

Participant 78

#### Student Preferences

This theme related to school counselors’ perceptions that young people like digital technology and, for various reasons, prefer doing activities and spending time on the Web. School counselors felt young people were more likely to engage with a Web-based service as it was delivered using a preferred medium. A total of 21% (n=30) school counselors reported that student preferences influenced their likelihood of referring, with most expressing this to be a positive effect (n=20). For example, as expressed by one of the participants *:*

...kids love online stuff, it’s their world.

Participant 6

Interestingly, however, a subset (7%, n=10) reported that some students had low levels of motivation to engage and complete online programs and that an online service would be unhelpful for these types of students. Further, a subset (7%, n=10) reported that student adherence to the service would be a risk to successful implementation.

#### Privacy and Confidentiality

This theme related to the primary concerns of privacy, anonymity, and confidentiality, such that school counselors were concerned that data provided over the Web may be mishandled or be accessed without authorization. A total of 7% (n=10) of the sample reported that issues relating to privacy and confidentiality influenced how comfortable they were with recommending or referring to a Web-based service. As one participant reported:

...potential for information to be lost or not given to right people.

Participant 22

A total of 8% (n=12) were uncomfortable with school counselors accessing students’ mental health data via the Web, and 78% (n=113) were also uncomfortable with teachers accessing students’ mental health data via the Web. For example, one participant stated:

Teachers have very different understandings of mental health concerns and levels of competence in managing them.

Participant 118

On the contrary, this theme also encompassed the advantages of Web-based interaction. Some participants reported positively:

E-MH [E-Mental Health] programs also provide confidentiality and students don't have to engage on a personal level when typing on a computer.

Participant 28

Overall, 14% (n=21) felt that issues relating to privacy and confidentiality posed a risk to the successful implementation of the service.

#### Informed Consent and Parental Contact

This theme related to school counselors’ concerns about collecting informed consent from students and how best to involve their parents. Although gaining informed consent was not expressed as a factor influencing school counselors’ referral or recommendation of a Web-based service, a small number of participants did identify it as a possible risk to successful implementation (4%, n=6). Participants’ responses confirmed that there are mixed feelings about students giving informed consent via the Internet and the need to inform others. For example, one of the participants responded:

The student would have to be well informed at the beginning of the process, so they are fully aware that some of their information may be passed on to others if required.

Participant 86

Nearly the entire sample (n=142) felt that parents should be involved at some point, with almost half (49%, n=72) reporting that parents should be informed when their child’s mental health scores were of clinical severity. Two-thirds (61%, n=89) felt that a phone call was the most ideal way to contact a parent, with only 25% (n=36) preferring an in-person consultation. Email (6%, n=9) and SMS (2%, n=3) were not popular methods of contacting parents. However, some expressed concerns with parental contact, as outlined by one participant:

...sometimes students don’t want parents to know they are seeking help.

Participant 20

## Discussion

### Principal Findings

This study aimed to understand school counselors’ roles in NSW secondary high schools, their level of comfort with Web-based resources and the proposed service, and to identify key barriers and facilitators to implementing such a service in the school setting. It was expected that this study would reveal key insights into the current context of school counseling, including the capacity of school counselors, as well as their likelihood of integrating a Web-based mental health service into their practice. On the basis of the study results, the average school counselor is a 43-year-old female, who is married or partnered, registered or completing registration as a psychologist, and has been practicing as a school counselor for more than 10 years. They are likely to be working full-time, but across two schools, with student populations of approximately 800 youth per school. The most common referral pathway to the school counselor was via student and teacher referrals, with only a minority occurring from initiation by school counselors themselves. School counselors reported undertaking a wide range of daily tasks with one-fifth of their time spent on administration. This confirms the multispecialist role of the school counselor [24].

The results of this study suggest that school counselors would benefit from additional mental health support in the school setting. One-third of school counselors reported that their workload was unmanageable. Work stress was reported to impact their health and well-being, and almost half had experienced burnout. Furthermore, one-third were providing care outside of school hours. Although this is consistent with previous results [22,27,47], it signifies that school counseling remains a demanding profession. Mental health issues, primarily anxiety and depression, were prominent among students, and despite the need, school counselors spent less than half of their time providing individual therapy. More support is necessary to reduce the personal burden of school counseling but also to increase school counselors’ capacity to service their students. Taken together, these findings provide evidence that clinical systems that increase efficiency would be highly favorable among school counselors. The levels of acceptability of the proposed Web-based service indicates that the stepped care model may provide the structured framework needed for improving the delivery of care, allowing greater detection of problems and more efficient allocation of resources.

Overall, one-third of the sample was entirely comfortable recommending the proposed Web-based service, and only 1 participant was not at all comfortable. Most of the school counselors believed that Web-based stepped care would be helpful to high school students, which is likely to be influenced by the fact that more than two-thirds of the sample already recommended Web resources to students. This is an important finding as it signifies support for the stepped care framework. It also indicates that school counselors are open to referring to Web-based interventions but have concerns that need to be alleviated before they will recommend or integrate the proposed service into their practice. This is not surprising, given the significant legal and moral obligations associated with providing mental health care to youth. The results show that some features of the proposed service, such as SMS or email monitoring, were more acceptable than others, such as mental health being classified by an automated process or the sharing of personal details. There was concern that responding in a timely manner to at-risk students would not be feasible. This is likely to reflect the high workload and low manageability within the sample. Future trials of the proposed service would need to monitor and measure the time taken to respond to at-risk students to quantify the feasibility of responding to real-time alerts.

Two-thirds of the sample reported that they often referred to external services and were mostly supported by these services. An external referral process is a key feature of the proposed Web-based service which is likely to be heavily utilized if widely implemented. Future trials of the service would need to ensure that external service providers are easily accessible for school counselors and that they have mechanisms of keeping in contact with the external providers. Background factors did not appear to influence the level of comfort. The qualitative results provide a significant level of insight into the issues that may need to be targeted and resolved for successful implementation. As already discussed, some of these relate to feasibility, but others, such as beliefs and level of knowledge, are specific to the individual counselor. These factors indicate that a significant amount of groundwork would need to take place, including training and demonstrations of the Web-based service, alongside case studies and evaluation data to increase the likelihood of school counselors recommending and referring to the service.

Finally, the results of this study outlined strengths of the proposed model. School counselors perceived that stigma, personal embarrassment/shame, and wanting to remain anonymous were significant barriers preventing students from seeking their help. This is consistent with other help-seeking literature [10], although only 5% of the current sample perceived that their own personal characteristics were a barrier to students seeking their help. Conversely, in their review, Gulliver et al [10] found that over one-third of studies reported that young people do not seek help because of concerns about characteristics of the provider. Despite the current sample not acknowledging this, students’ help-seeking is likely to be impacted by provider characteristics. The proposed service may circumvent this issue, as students are able to self-refer. In addition, allowing teacher-referrals may increase uptake of the service, as some students report more trusting and responsive relationships with teachers [48]. Future trials of the proposed service would benefit from measuring changes in student attitudes toward seeking help from different sources, as well as actual help-seeking behavior, specifically from the school counselor. This would help to confirm whether the service is effective for increasing access to and uptake of mental health care.

### Limitations

Despite the depth of insight, the current sample was drawn exclusively from one state within Australia (NSW). Views may differ among international samples, where the role of school counseling may be different. In addition, the self-report data are subjective and subject to misrepresentation [49]. The volunteer sample was not representative, and it was not possible to estimate bias resulting from this and the questions asked may have precluded factors that influence school counselors’ views.

### Conclusions

Overall, this study confirms that greater support and resources are needed to facilitate what is already a challenging and emotionally demanding role for school counselors. The school counselors surveyed were supportive of the proposed stepped care service, and their insight has informed the service development in several ways. Key next steps would be a demonstration and trial of the service in the school environment, alongside a formal evaluation of its usefulness and effectiveness, with outcomes assessed for both students and school counselors.

## Appendix

Multimedia Appendix 1 Online survey.

## Abbreviations

ATSI: Aboriginal or Torres Strait Islander
EBPAS: evidence-based practice attitude scale
GP: general practitioner
NSW: New South Wales
SD: standard deviation
SMS: short message service
