# Supplementary material for: School Counselors’ Perspectives of a Web-Based Stepped Care Mental Health Service for Schools: Cross-Sectional Online Survey
Source: JMIR Ment Health. 2017 Nov 20;4(4):e55. doi: 10.2196/mental.8369 (PMC5715202; doi:10.2196/mental.8369)
Supplement: Multimedia Appendix 1 [file mental_v4i4e55_app1.pdf]

ONLINE SURVEY FOR SCHOOL COUNSELLORS

PART 1: DEMOGRAPHICS

1. How old are you?
2. What is your gender?
    a. Male
    b. Female
    c. Other
3. Do you identify as Aboriginal or Torres Strait Islander?
    a. Yes
    b. No
    c. I'd rather not say
4. Were you born in Australia?
    a. Yes
    b. No (please state which country you were born in):
5. Please provide the postcode of your home address:
6. How many dependent children do you have under the age of 18 years?
7. What is your relationship status?
    a. Single
    b. Married or partnered
    c. Divorced or separated
    d. Widowed
    e. Other______________________________
8. What is the highest level of education you have completed?
    a. Year 11 or below
    b. Year 12 or equivalent
    c. TAFE Certificate III or IV
    d. Bachelor’s Degree
    e. University Graduate Diploma
    f. University Postgraduate Degree
    g. Other_______________________________
9. Are you a registered Psychologist?
    a. Yes
    b. No
    c. I am currently undertaking registration
10. For how many years have you been working as a School Counsellor?
11. How many schools do you currently work in as a School Counsellor?
12. Since you began working as a School Counsellor, in how many different schools have you worked?

PART 2: CURRENT SCHOOL

Please answer the following questions in regards to the school at which you are currently employed. If you are currently employed at more than one school, please choose the school at which you primarily work (i.e., where spend the most time):

13. How would you best describe your current role?
    a. School Counsellor
    b. Mobile School Counsellor
    c. School Psychologist
    d. Guidance Officer
    e. Student Support officer

f. Head Techer Wellbeing
g. Other

14. Is your school:
    a. Government
    b. Government (selective or selective stream)
    c. Independent
    d. Religious
    e. Other
15. Is your current role full-time, part-time or casual?
    a. Full-time
    b. Part-time
    c. Casual
16. Is your school:
    a. Co-educational
    b. Single sex
17. Approximately how many students are enrolled in your school?
18. What is the postcode of the school at which you are currently employed?
19. What is the most common way that students are referred to you?
    - Student refers themselves
    - School Counsellor initiates contact
    - Teacher referrals
    - Year Advisor referrals
    - Parent referrals
    - Other
20. On an average working day, approximately how many sessions do you have with students?
21. Of these sessions, approximately how many are related to mental health concerns?
    1. None at all
    2. Some of them
    3. Half of them
    4. Most of them
    5. All of them
22. Please choose which mental health issues are most prevalent among students in your current school?
    - Depression/ low mood
    - Anxiety/ excessive worry
    - Conduct disorder/ behavioural problems
    - Family issues
    - Self-harm
    - Drug/ alcohol misuse
    - Eating disorders
    - Attention and hyperactivity related issues
    - Autism related issues
    - Psychosis
    - Relationship problems
    - Bullying
    - Stress
    - Truancy
    - Other
23. Of these, which mental health issue do you consider to be the most significant at your current school?

24. On a scale of 1 - 5, how comfortable do you think students are in approaching you to talk about their mental health?
    1. Not at all
    2. Slightly
    3. Somewhat
    4. Moderately
    5. Entirely
25. Which barriers do you think stop students from approaching you for help? (You may select more than one)
    - Stigma from others
    - Lack of understanding/ knowledge about what the service can offer Wanting to remain anonymous
    - Personal embarrassment or shame
    - Distrust in the school
    - Cultural perceptions of mental health/ illness
    - Personal characteristics of the counsellor
    - Concern about confidentiality
    - Other
26. What percentage (%) of your workload is occupied with the following activities:
    - Individual Therapy
    - Group Therapy
    - Administrative tasks
    - Psychometric assessments
    - Professional Development
    - Personal or Group Supervision
    - Crisis management
    - Risk assessment for suicide or self-harm
    - Other
    - Total
27. How often do you provide students with your personal contact details?
    - Never
    - Rarely
    - Sometimes
    - Often
    - Always
28. How often do you find yourself providing care outside of school hours?
    - Never
    - Rarely
    - Sometimes
    - Often
    - Always
29. How often do you refer students to external services?
    - Never
    - Rarely
    - Sometimes
    - Often
    - Always
30. How supported do you feel by external services?
    1. Not at all

2. Slightly
3. Somewhat
4. Moderately
5. Completely

31. How often do you engage with the parents/ guardians of the students that you see?
    - Never
    - Rarely
    - Sometimes
    - Often
    - Always
32. How supported do you feel by the parents/ guardians of the students you see?
    1. Not at all
    2. Slightly
    3. Somewhat
    4. Moderately
    5. Entirely
33. How supported do you feel by other teachers?
    1. Not at all
    2. Slightly
    3. Somewhat
    4. Moderately
    5. Entirely
34. On a scale of 1 - 5, how manageable is your current workload?
    1. Not at all
    2. Slightly
    3. Somewhat
    4. Moderately
    5. Entirely
35. On a scale of 1 - 5, to what extent do you think that work stress impacts on your health and wellbeing?
    1. Not at all
    2. Slightly
    3. Somewhat
    4. Moderately
    5. Entirely
36. How often do you engage in deliberate self-care?
    - Never
    - Rarely
    - Sometimes
    - Often
    - Always
37. Have you ever experienced "burnout" (physical and/or emotional exhaustion resulting from work) related to your role as a school counsellor?
    - Yes
    - No
    - Unsure
    - I'd rather not say
38. Overall, how satisfied are you with the way your school addresses mental health for its students?
    1. Not at all
    2. Slightly

3. Somewhat
4. Moderately
5. Entirely

Please indicate the extent to which you agree with each of the following statements:

39. I like to use new types of therapy/ interventions to help my clients.
    1. Not at all
    2. To a slight extent
    3. To a moderate extent
    4. To a great extent
    5. To a very great extent
40. I am willing to try new types of therapy/ interventions even if I have to follow a treatment manual.
    1. Not at all
    2. To a slight extent
    3. To a moderate extent
    4. To a great extent
    5. To a very great extent
41. I am willing to use new and different types of therapy/ interventions developed by researchers.
    1. Not at all
    2. To a slight extent
    3. To a moderate extent
    4. To a great extent
    5. To a very great extent
42. I would try a new therapy/ intervention even if it were different from what I am used to doing.
    1. Not at all
    2. To a slight extent
    3. To a moderate extent
    4. To a great extent
    5. To a very great extent

How often do you use, recommend, or refer students to the following e-mental health programs or resources in your work?

1. Mental health information and/or psycho-education sites
    - Never
    - Sometimes
    - Often
    - Always
2. Peer support (e.g., online forums, blogs, personal journals)
    - Never
    - Sometimes
    - Often
    - Always
3. Mobile phone apps
    - Never
    - Sometimes
    - Often
    - Always

4. Symptom focused (e.g., online CBT programs)
    - Never
    - Sometimes
    - Often
    - Always
5. Online counselling services
    - Never
    - Sometimes
    - Often
    - Always
6. Access services by videoconferencing (e.g., Skype)
    - Never
    - Sometimes
    - Often
    - Always

43. Why/ or why not do you use, recommend, or refer students to the above e-mental health programs or resources in your work? (Short answer response)

PART 3: SERVICE EVALUATION

Online Stepped Care Service The Black Dog Institute has designed a new online service to address mental health concerns (primarily anxiety and depression) among high school students. The service is based on the "Stepped Care" model for depression, and is designed to be delivered in the school setting, in partnership with the Black Dog Institute. The service will involve the young person visiting a website, registering their details, and completing an online screening questionnaire to assess their current mental health needs. The young person will then have access to a range of information and activities via the website that will improve their wellbeing. This service is comprised of 5 stages. The final part of this survey will briefly describe and seek feedback about each stage.

Stage 1: Recommendation A student can be recommended to go online and try this service by a teacher, school counsellor, or another health professional. A student can also visit the online service without a recommendation.

44. How comfortable would you be recommending a student to try an online service for their mental health?
    1. Not at all
    2. Slightly
    3. Somewhat
    4. Moderately
    5. Entirely
45. How comfortable would you be with a teacher recommending a student to try an online service for their mental health?
    1. Not at all
    2. Slightly
    3. Somewhat
    4. Moderately
    5. Entirely
46. How comfortable would you be with another health professional (such as a doctor) recommending a student to try an online service for their mental health?

    1. Not at all
    2. Slightly
    3. Somewhat
    4. Moderately
    5. Entirely
47. Do you have any comments about the recommendation process? (Short answer response)

Stage 2: Sign-up and Questionnaire. When a student visits the online service, they are asked to create an account in which they have to provide a username and password, as well as their name, date of birth, email address, mobile phone number, and school. They may also be asked to give their parents' contact details such as name, phone number, and email address. When a student signs in, they will then be asked to answer a series of questions about their mental health including depression, anxiety, suicidal ideation, self-harm, alcohol and drug use.

48. How comfortable would you be with a student providing these personal details (name, date of birth, email address, etc.) to an online mental health service?
    1. Not at all
    2. Slightly
    3. Somewhat
    4. Moderately
    5. Entirely
49. How comfortable would you be with a student answering questions about their mental health over the internet?
    a. Not at all
    b. Slightly
    c. Somewhat
    d. Moderately
    e. Entirely
50. How comfortable would you be with the school counsellor having access to all students' responses (within a particular school) to the mental health questions?
    a. 1. Not at all
    b. 2. Slightly
    c. 3. Somewhat
    d. 4. Moderately
    e. 5. Entirely
51. How comfortable would you be with teachers seeing their students' responses to the mental health questions?
    a. 1. Not at all
    b. 2. Slightly
    c. 3. Somewhat
    d. 4. Moderately
    e. 5. Entirely
52. How comfortable would you be with another health professional seeing students' responses to the mental health questions?
    a. 1. Not at all
    b. 2. Slightly
    c. 3. Somewhat
    d. 4. Moderately
    e. 5. Entirely
53. Do you have any other comments about the sign-up process? (Short answer response)

Stage 3: Stepping. Based on a student's responses to these questions, the program uses a formula to decide which “step” of mental healthcare they need. These are mathematical formulas that are based on scientific and clinical evidence. There are 4 steps. Step 1 is for students that are not having any mental health issues. Step 2 is for students that are having mild symptoms, Step 3 is for students that are having moderate symptoms, and Step 4 is for students that are having severe symptoms.

54. How comfortable would you be with a computer program like this deciding which step of care a student needs?
    a. Not at all
    b. Slightly
    c. Somewhat
    d. Moderately
    e. Entirely

Once a student has been stepped, they then receive access to a range of information and activities that are suitable for their mental health needs. These are also based on scientific and clinical evidence. Students who are at Steps 1 and 2 (the nil - mild symptom levels) can access the information and complete the activities over the internet, in their own time. In addition to this access, students who are at Steps 3 and 4 (the moderate - severe levels) will automatically be linked in with a face-to-face service. One possible service is the school counsellor. In this example, the school counsellor would be notified to arrange a face-to-face session as soon as possible.

55. How comfortable would you be with a student receiving care and treatment for mental health problems via an online program?
    a. Not at all
    b. Slightly
    c. Somewhat
    d. Moderately
    e. Entirely
56. In your opinion, how feasible would it be for the school counsellor to initiate contact and organise a face- to-face session (within 24-48 hours) with the students who are allocated to Steps 3 and 4? (Research suggests this would most likely be approximately 15% of all students; so in a school of 600, this would be 90 students).
    a. Not at all
    b. Slightly
    c. Somewhat
    d. Moderately
    e. Entirely
57. How comfortable would you be with a student who is at Step 3 or Step 4 being contacted by an external psychologist over the phone for psychological support?
    a. Not at all
    b. Slightly
    c. Somewhat
    d. Moderately
    e. Entirely
58. Do you have other comments about Stage 3 - the stepping process? (Short answer response)

Stage 4: Monitoring. Every 14 days, the student will receive an email or text message that briefly checks how they are doing.

59. How comfortable are you with the idea of a student being sent emails or text messages to check in on their mental health?
    a. 1. Not at all
    b. 2. Slightly
    c. 3. Somewhat
    d. 4. Moderately
    e. 5. Entirely
60. Based on this monitoring, those students who are not doing well will be ‘stepped up’ to the higher level of care. Monitoring will continue for the duration of the school year.
61. What do you think should happen if a student isn’t getting better or is getting worse? (Short answer response)
62. What do you think should happen if a student reports they are suicidal or engaging in self-harm? (Short answer response)
63. Do you have any other comments about Stage 4 - the monitoring process? (Short answer response)

Stage 5: Feedback. Using the monitoring system, the program has the ability to send feedback to nominated individuals such as a parent, a school counsellor, a teacher or another health professional.

64. Overall, do you think that this type of program would be helpful to high school students?
    a. Yes
    b. No
    c. Unsure
65. Would you prefer that this type of program is offered completely separate from the school?
    a. Yes
    b. No
    c. Unsure
66. Can you think of any risks associated with a model like this? (Short answer response)
67. Do you have any other comments? (Short answer response)
